# Novel Anti-Infective Compounds from Marine Bacteria

**DOI:** 10.3390/md8030498

**Published:** 2010-03-05

**Authors:** Hafizur Rahman, Brian Austin, Wilfrid J. Mitchell, Peter C. Morris, Derek J. Jamieson, David R. Adams, Andrew Mearns Spragg, Michael Schweizer

**Affiliations:** 1 School of Life Sciences, Heriot-Watt University, Riccarton, Edinburgh EH14 4AS, Scotland, UK; E-Mails: h.rahman@hw.ac.uk (H.R.); w.j.mitchell@hw.ac.uk (W.J.M.); p.c.morris@hw.ac.uk (P.C.M.); d.j.jamieson@hw.ac.uk (D.J.J.); m.schweizer@hw.ac.uk (M.S.); 2 Institute of Aquaculture, University of Stirling, Stirling, FK9 4LA, Scotland, UK; 3 Department of Chemistry, School of Engineering and Physical Sciences, Heriot-Watt University, Riccarton, Edinburgh EH14 4AS, Scotland, UK; E-Mail: d.r.adams@hw.ac.uk (D.R.A.); 4 Aquapharm Biodiscovery Limited, European Centre for Marine Biotechnology, Dunstaffnage Marine Laboratory, Oban, Argyll PA37 1QA, Scotland, UK; E-Mail: andrew@aquapharm.co.uk (A.M.S.)

**Keywords:** marine bacteria, MRSA, VRE, hospital superbugs, new scaffolds, antibiotic resistance, nosocomial infection

## Abstract

As a result of the continuous evolution of microbial pathogens towards antibiotic-resistance, there have been demands for the development of new and effective antimicrobial compounds. Since the 1960s, the scientific literature has accumulated many publications about novel pharmaceutical compounds produced by a diverse range of marine bacteria. Indeed, marine micro-organisms continue to be a productive and successful focus for natural products research, with many newly isolated compounds possessing potentially valuable pharmacological activities. In this regard, the marine environment will undoubtedly prove to be an increasingly important source of novel antimicrobial metabolites, and selective or targeted approaches are already enabling the recovery of a significant number of antibiotic-producing micro-organisms. The aim of this review is to consider advances made in the discovery of new secondary metabolites derived from marine bacteria, and in particular those effective against the so called “superbugs”, including methicillin-resistant *Staphylococcus aureus* (MRSA) and vancomycin resistant enterococci (VRE), which are largely responsible for the increase in numbers of hospital acquired, *i.e.*, nosocomial, infections.

## 1. Introduction

**Figure 1:**
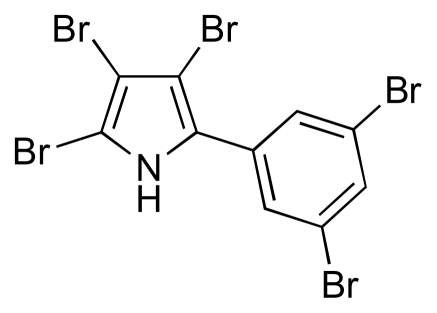
Structure of pentabromopseudilin.

The search for new antibiotics is an important element in the fight against the threat posed by the increase in the number of infections caused by antibiotic-resistant pathogens. Literature reviews show that most antibiotics used today originate from a small set of scaffold compounds, with many other antibiotics being synthetically derived from them [1]. Resistance of pathogenic bacteria toward antibiotics is certainly increasing, but the rate of discovery and development of new and effective antibiotic compounds is declining [2,3]. Among the clinically used antibiotics, over two thirds have been discovered from natural sources or are the semi-synthetic derivatives of natural antibiotics [4]. Until now, the dominant effort to discover new antibiotics has involved the terrestrial environment whereas, in comparison, little attention has been given to marine microbial metabolites. Indeed, by the end of 2008, only ~3000 microbial metabolites had been reported from marine sources, which is far less than the number of compounds isolated from other natural sources [5,6]. A long-held belief of many scientists that seawater contained few microbes was the main reason for overlooking the secondary metabolites from marine micro-organisms. Pentabromopseudilin [7] (Figure 1), which was the first marine antibiotic to be recognized, was discovered in 1966. Yet, the majority of natural antibiotics have been found in soil-borne actinomycetes, perhaps because they can be readily and easily recovered and cultured, as well as the perception that they dominate as antibiotic-producers. The marine environment with its diverse microflora is likely to be a very promising source of novel antibiotic producers. Indeed, a new anti-folate scaffold, termed abyssomicin, was recently isolated from a marine *Verrucosispora*, which is a rare actinomycete genus belonging to the family Micromonosporaceae [8]. Thus, it is reasoned that marine microbial metabolites offer the prospect of potential candidates for new drug discovery programmes [9]. Indeed, there are already some detailed reviews on marine natural products [10,11] with some focusing on compounds for infectious diseases, examples of which include products with antituberculosis [12], antiviral [13], antiparasitic [14], antinematodal [15] and antifungal [16] activity. Consequently, the main objective of this article is to highlight the importance of marine bacteria as a potential source of new compounds with activity against hospital-acquired infectious diseases, especially methicillin resistant *Staphylococcus aureus* (MRSA) and vancomycin resistant enterococci (VRE).

## 2. Anti-methicillin-resistant *Staphylococcus aureus* (MRSA) and Anti-vancomycin-resistant Enterococci (VRE) Compounds Derived from Marine Bacteria

**Figure 2:**
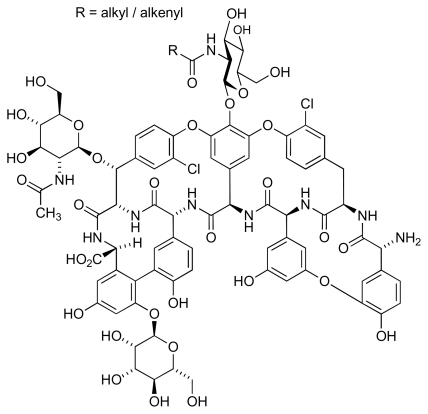
Structure of teicoplanin.

**Figure 3:**
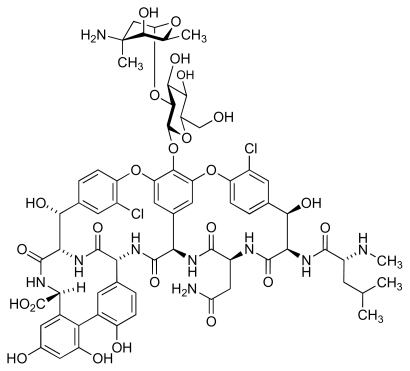
Structure of vancomycin.

MRSA infection is considered to be one of the main causes of death among the hospital-acquired infectious diseases, and has led to many thousands of deaths and additional expenditure estimated as 3–4 billion dollars for health care in the USA [17]. MRSA is a Gram-positive spherical organism, often occurring as clusters of cells. The organism can rapidly mutate to acquire resistance to antibacterial agents [18,19] and is highly resistant to commercially available antibiotics, with the exception of teicoplanin (Figure 2) and vancomycin (Figure 3) [20,21]. Worryingly, recent studies have identified the existence of vancomycin-resistant [22,23] and teicoplanin-resistant *S. aureus* [24,25]. Thus, there is urgency for the development of new drugs effective against MRSA. The first report of vancomycin resistance was published in 1988, and described an isolate of *Enterococcus faecium* [26]. Unfortunately, the number of vancomycin-resistant isolates of *S. aureus* or *Enterococcus* (VRSA and VRE) causing disease is steadily increasing, but they are as yet less prevalent than MRSA [27]. However, treatment of VRE is more difficult as there are no effective alternative antibiotics available [28].

**Figure 4:**
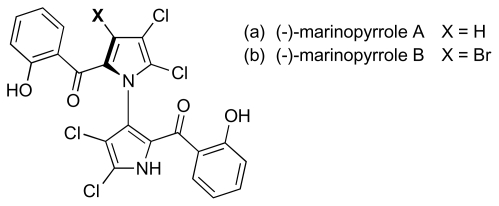
Structure of marinopyrroles A and B.

Cultivation of a marine *Streptomyces* sp. (CNQ-418) recently yielded two densely halogenated metabolites, marinopyrroles A and B (Figure 4), which showed potent activity against MRSA [minimum inhibitory concentrations (MIC) ≤ 2 μM] as well as cytotoxicity against a human cancer cell line (HCT-116) [29]. Like pentabromopseudilin, marinopyrroles A and B possess halogenated pyrrole subunits, although the N,C_2_-linked bispyrrole motif that they embody has not previously been observed in natural product construction, and they constitute a structurally novel class of antibiotic. Notably the biaryl bond in these compounds, flanked by four *ortho* substituents, establishes an axis of chirality, and the fact that both structures were isolated as single (−)-atropo-enantiomers may suggest a key enzyme-mediated pyrrole coupling in their biosynthesis. Studies with marinopyrrole A showed that the biaryl linkage is configurationally stable at room temperature, although the compound can be racemized at elevated temperatures to yield the non-natural (+)-enantiomer. Interestingly, the latter had comparable activity against MRSA [MIC 0.31 μM *versus* 0.61 μM for the (−)-enantiomer]. However, the mechanism of action of these compounds has yet to be defined.

**Figure 5:**
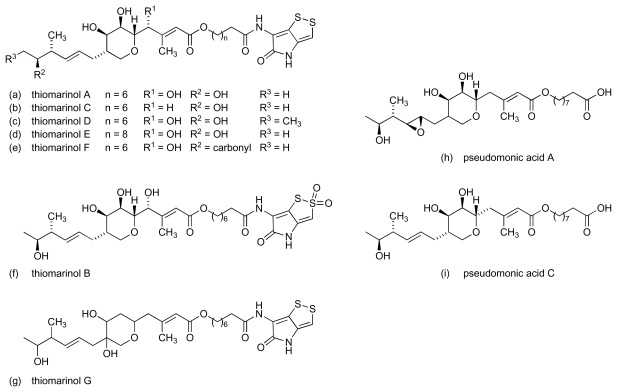
Structure of thiomarinols A-G and pseudomonic acids A and C.

Another marine bacterium, *Alteromonas rava* SANK 73390, has been shown to produce several sulphur-containing secondary metabolites, thiomarinols A-G (Figure 5a–g), with broad spectrum activity against Gram-positive and Gram-negative species [30–33]. The thiomarinols are especially active against Gram-positive bacteria and have pronounced activity against MRSA, in particular (MICs ≤ 0.01 μg/mL). Their antibacterial mode of action is reportedly mediated by inhibition of bacterial isoleucyl-transfer RNA synthetase, the same mechanism as described [34] for the structurally related pseudomonic acids (e.g., pseudomonic acids A and C, Figure 5h/5i), which were originally isolated from *Pseudomonas fluorescens* NCIMB 10586 [35] and are the main constituents in the topical antibiotic, mupirocin. Studies with thiomarinols A, B and D revealed pronounced specificity for inhibition of isoleucyl-tRNA synthetase over the valyl- and leucyl-tRNA synthetases [30].

**Figure 6:**
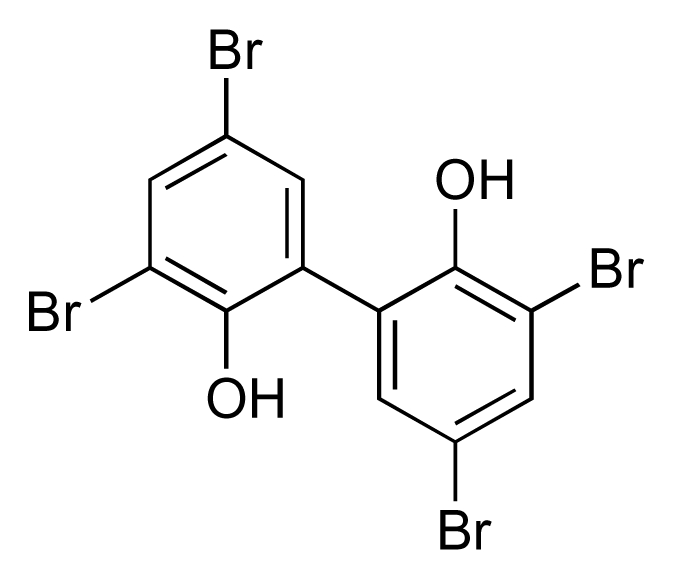
Structure of MC21-A.

**Figure 7:**
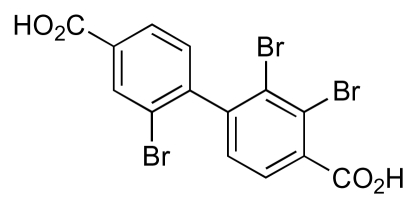
Structure of MC21-B.

Two halogenated biphenyl secondary metabolites, MC21-A (Figure 6) and MC21-B (Figure 7), were isolated from the marine bacterium *Pseudoalteromonas phenolica* O-BC30^T^, and demonstrated activity against ten clinical isolates of MRSA with MICs of 1–4 μg/mL [36]. Significantly the level of activity of these compounds was comparable to that of vancomycin (MICs of 0.25–2 μg/mL). MC21-A had no cytotoxic effect on human normal fibroblasts, and exhibited significant bactericidal activity, which was also comparable with that of vancomycin. In contrast, MC21-B (Figure 7) was found to be cytotoxic to human normal dermal fibroblasts and human leukaemia cells [37]. Subsequent studies determined that MC21-A killed MRSA cells by interfering with the permeability of the cell membrane [38]. MC21-A also showed high activity against *Enterococcus seriolicida*, *Enterococcus faecium*, and *Enterococcus faecalis*.

**Figure 8:**
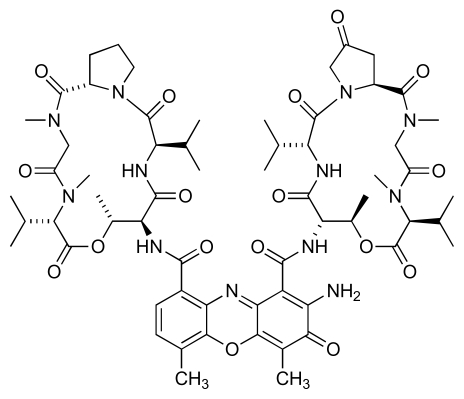
Structure of actinomycin V.

Actinomycetes isolated from samples of Korean marine silt have also been screened for production of anti-MRSA natural products. During this work a compound (AM3) with potent anti-MRSA activity was isolated from the culture broth of a *Streptomyces* strain, AM045 [39]. The level of activity exhibited by the compound (MIC: 0.1~0.4 μg/mL) was higher than that of vancomycin or teicoplanin, but the structure was subsequently identified as actinomycin V (Figure 8) [39].

**Figure 9:**
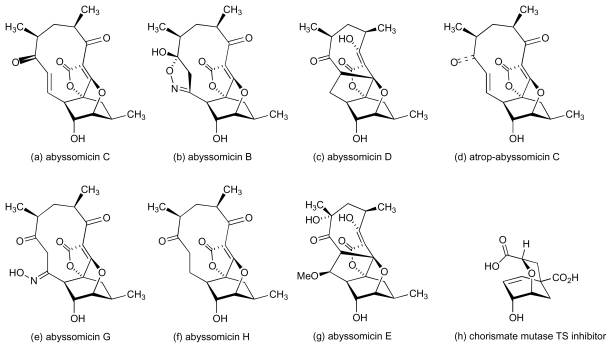
Structure of abyssomicins.

Culture extracts from two hundred marine and terrestrial actinomycetes were recently screened in a search for novel antibacterial compounds that specifically disrupt biosynthesis of *para*-aminobenzoic acid (*p*ABA), a key intermediate *en route* to tetrahydrofolate. These screens led to the discovery of abyssomicin C (Figure 9a), a complex polyketide-type compound isolated from a marine strain, *Verrucosispora* AB-18-032, which was obtained from a sediment sample collected in the Sea of Japan at a depth of 289 m [40,41]. Abyssomicin C showed good inhibitory activity against MRSA and VRSA (MIC 4–13 μg/mL), though two analogues (abyssomicins B and D, Figure 9b/c), which were isolated at the same time, lacked any antibacterial activity. The activity of abyssomicin C, the first natural compound reported to inhibit biosynthesis of *p*ABA, prompted attempts to develop synthetic routes to the compound and these culminated in successful total syntheses [42–44]. Remarkably, conformational constraints in the macrocyclic ring led to the issue of two isolable atropisomeric states from the total synthesis undertaken by Nicolaou’s group [42], abyssomicin C and atrop-abyssomicin C (Figure 9a and 9d respectively); the latter isomer was somewhat more potent in its activity against MRSA. Although initially thought to be a non-natural isomer, atrop-abyssomicin C was subsequently also recovered from cultures *Verrucosispora* AB-18-032 together with a further two analogues, abyssomicins G and H (Figure 9e and 9f), that lacked antibacterial activity [40]. One other member of this natural product family, abyssomicin E (Figure 9g), has also been isolated but from a terrestrial *Streptomyces* sp. (HKI0381) [45]. Also, this compound did not exhibit antibacterial activity. The antibacterial activity of abyssomicin C and its atropisomer is therefore strongly dependent on the presence of the Michael acceptor unit within the macrocyclic ring, and the absence of this feature in abyssomicins B, D, E, G and H accounts for their lack of activity. The importance of the Michael acceptor unit is further underlined by the finding that reduction of the enone carbonyl in abyssomicin C ablates activity [46]. Blockade of *p*ABA biosynthesis by abyssomicin C arises from inhibition of enzymes that process chorismate [41,47], and it has been noted [41] that the abyssomicin oxabicycloctane system bears a striking resemblance to known transition-state analogue inhibitors of chorismate mutase (Figure 9h) [48].

**Figure 10:**
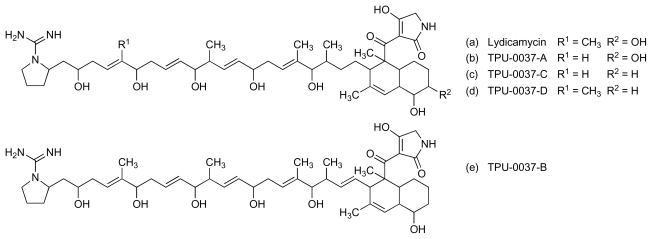
Structure of lydicamycin and TPU-0037-A to D.

Culture extracts of another marine actinomycete, *Streptomyces platensis* (TP-A0598), yielded four secondary metabolites, TPU-0037-A to D (Figure 10b–e), that exhibited activity against MRSA with MICs in the range 3–13 μg/mL [49]. These compounds are close congeners of an established antibiotic, lydicamycin (Figure 10a), that was originally isolated from cultures of *Streptomyces lydicus* taken from a soil sample [50,51]. Lydicamycin exhibited anti-MRSA activity with a MIC of 6 μg/mL [49].

**Figure 11:**
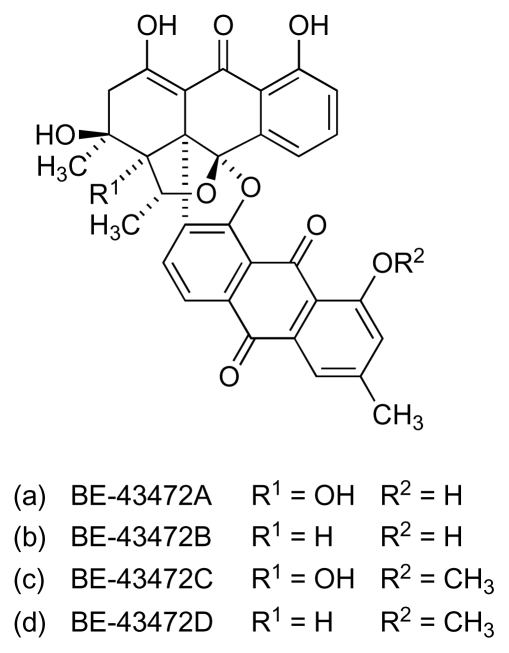
Structure of bisanthraquinones BE-43472A to D.

Four unusual, streptomycete-derived bisanthraquinone compounds with antitumor-antibiotic activity, BE-43472A to D (Figure 11a–d), were first claimed in a Japanese patent in 1996 [52]. A decade later, the compounds were also reported as natural products from a marine *Streptomyces* strain (N1-78-1) isolated from cultured cells of an unidentified cyanobacterium (URI strain N36-11-10), itself collected from a Puerto Rican ascidian, *Ecteinascidia turbinata* [53,54]. In this latter study, the relative stereochemistry of the compounds was defined and the compounds were determined to have potent antibacterial activity against MRSA and VRE, as well as cytotoxic activity against HCT-116 cells. BE-43472B exhibited the most potent activity against MRSA and VRE with MICs in the range 0.11–0.45 μM, and a minimum bactericidal concentration against a number of clinical MRSA isolates in the range 0.91–3.6 μM; 29 μM against VRE [54]. In the initial reports [53,54] the structures of BE-43472A to D were depicted with antipodal stereochemistry to that shown in Figure 11. Unambiguous assignment of the absolute stereochemistry was subsequently facilitated, however, with the successful total synthesis of both enantiomers of BE-43472B [55,56]. Interestingly the unnatural (−)-BE-43472B enantiomer was found to exhibit comparable antibacterial activity to that of the natural (+)-enantiomer (Figure 11b).

**Figure 12:**
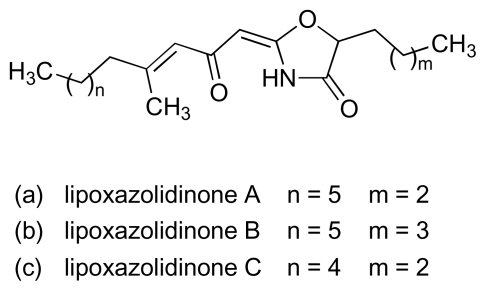
Lipoxazolidinones A to C.

**Figure 13:**
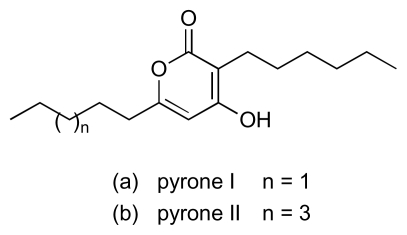
Pyrones I and II.

**Figure 14:**
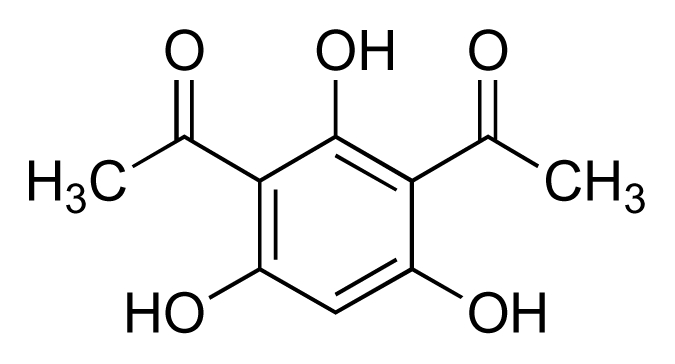
2,4-Diacetylphloroglucinol.

**Figure 15:**
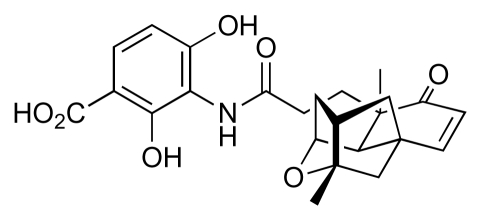
Platensimycin.

A number of structurally less complex natural products with activity against MRSA and VRE have also been isolated from cultures of marine bacteria since 2000. Thus, a new class of 2-alkylidene-4-oxazolidinone, exhibiting an unprecedented antibiotic pharmacophore, was isolated from a marine actinomycete (NPS8920) [57,58]. A series of three compounds in this class, lipoxazolidinones A to C (Figure 12), was isolated, with the most potent activity against MRSA and VRE exhibited by congener A (MICs: 1–2 μg/mL). Absolute stereochemistry for the lipoxazolidinones has yet to be determined. The ubiquitous bacterial type II fatty acid synthase (FASII) is essential for bacterial viability. A screening programme designed to discover new antibiotics that block bacterial fatty acid biosynthesis by targeting the FASII complex recently identified two lipophilic *α*-pyrones from a marine *Pseudomonas* sp. (F92S91) (Figure 13) that possessed activity against MRSA, VRE and other bacteria [59]. Pyrone I (Figure 13a) was the more potent of the two compounds, exhibiting MICs in the range of 2–4 μg/mL and 2–64 μg/mL against MRSA and VRE strains, respectively. Investigations with *Bacillus subtilis* showed that pyrone I interferes with membrane function and non-specifically inhibits incorporation of radio-labeled precursors into RNA, DNA and protein. Another marine *Pseudomonas* sp. (strain AMSN), which was isolated from the surface of a red alga (*Ceratodiction spongiosum*), produced 2,4-diacetylphloroglucinol (DAPG; Figure 14) which exhibited potent anti-MRSA, anti-VRSA and anti-VRE activities against a panel of clinical isolates with MICs in the range 1–8 μg/mL [60–62]. This simple, robust compound, which is stable at temperatures up to 70 °C and across a wide pH range (pH 2–7), lacked acute toxicity in mice at levels of up to 100 mg/kg. The inhibition of cellular lipid biosynthesis is thought to underpin the antibacterial activity of another polyphenolic antibiotic, platensimycin (Figure 15), disclosed by Merck in 2006 [63]. The Merck group isolated platensimycin from cultures of *Streptomyces platensis*, recovered from soil samples, although the compound had previously been isolated from marine bacteria in work that was unpublished [64]. Platensimycin demonstrated broad spectrum activity against Gram-positive bacteria and was very effective against antibiotic-resistant strains, including MRSA, vancomycin-intermediate-resistant *S. aureus* (VISA) and VRE, successfully eradicating MRSA infection in mice. Platensimycin reportedly inhibits the elongation condensing enzymes, FabF/B, that are essential components of fatty-acid biosynthesis in FASII and are highly conserved in pathogens [63]. A recent report, however, calls into question the attractiveness of FASII as target pathway for development of next generation antibiotics [65].

**Figure 16:**
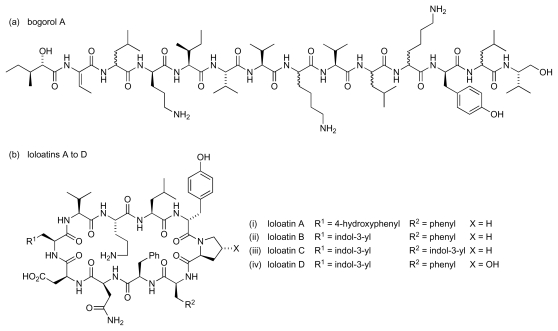
Peptidic antibiotics derived from marine bacteria.

Peptides also feature among the naturally occurring compounds from marine bacteria that possess activity against antibiotic-resistant pathogens (Figure 16). Thus, a novel peptide antibiotic, bogorol A (Figure 16a), which was isolated from a marine *Bacillus,* was found to possess good activity against MRSA and VRE (MICs of 2 μg/mL and 10 μg/mL respectively) [66]. Bogorol A exhibited more modest activity against *Escherichia coli* (35 μg/mL), *Burkholderia cepacia* (200 μg/mL), *Pseudomonas aeruginosa* (200 μg/mL) and *Candida albicans* (200 μg/mL). Significant features of the structure include unusual terminal subunits, as the structure contains both a reduced *C*-terminal valine modification and an *N*-terminal (*R*)-hydroxy acid subunit. Chiral HPLC analysis of the derivatised hydrolysate of bogorol A was used to determine the stereochemistry of constituent amino acids. As the compound was found to possess both L- and D-leucine as well as L- and D-lysine, assignment of absolute configuration to all of the stereogenic centres in the structure was not possible. However, bogorol A may provide a basis for a combinatorial approach to new agents with activity against MRSA and VRE. Four cyclic decapeptide antibiotics, loloatins A to D (Figure 16b), were isolated from another marine *Bacillus* sp. MK-PNG-276A, which was collected from the Great Barrier Reef off Papua New Guinea [67–69]. Loloatins A-D exhibited *in vitro* antimicrobial activity against MRSA (MICs respectively: 4 μg/mL, 2 μg/mL, 0.5 μg/mL and 8μg/mL) as well as VRE (MICs: 4 μg/mL, 2 μg/mL, 1 μg/mL and 8 μg/mL). A number of reports have subsequently discussed the solution phase conformation of the loloatins and described the synthesis of loloatin anlogues [70–74].

**Figure 17:**
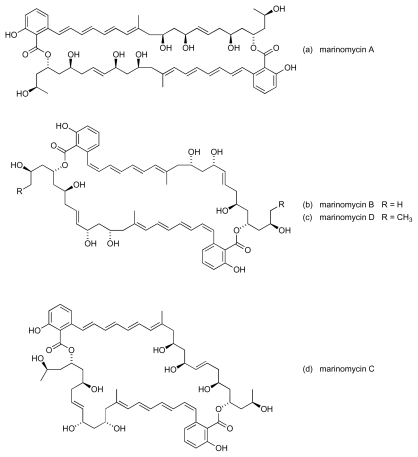
Structure of marinomycins.

A series of bioactive polyene-polyols, marinomycins A to D (Figure 17), was isolated from a newly discovered marine actinomycete genus named *Marinispora* [75]. Congeners A, B and D were found to possess a symmetrical macrodiolide construction, whereas marinomycin C was unsymmetrical. Marinomycin A exhibited the most potent antibacterial activity of the series, with an *in vitro* MIC of 0.13 *μ*M against both MRSA and VRE; marinomycins B to D were active against MRSA (MIC ~0.25 *μ*M in each case) but these three congeners showed no significant activity against VRE. The structure of marinomycin A is reminiscent of other well-known polyene antifungal-antibiotics, such as amphotericin B that form clusters within membranes, creating ion channels. However, a lack of significant antifungal activity in the marinomycins suggests that these new macrolides are not membrane active but function through an, as yet, unidentified mechanism [75].

**Figure 18:**
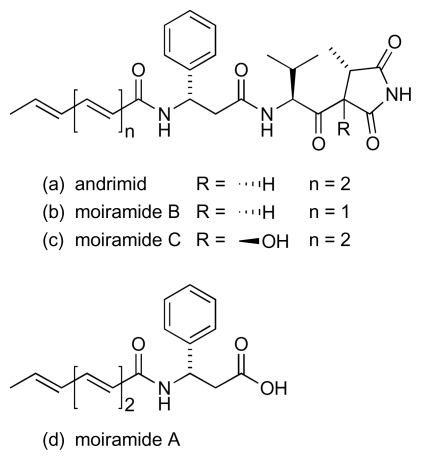
Andrimid and moiramides A–C.

**Figure 19:**
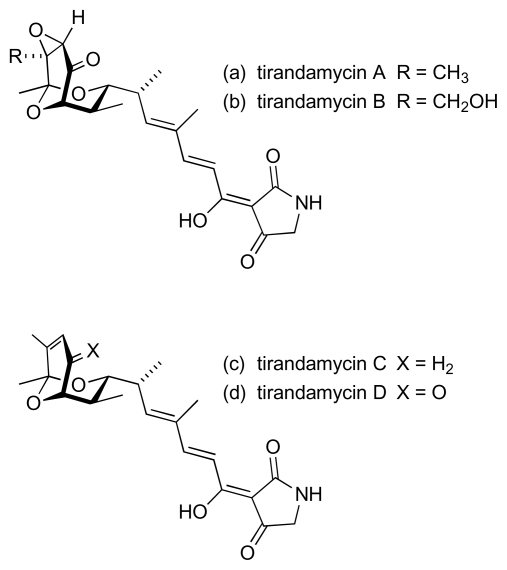
Tirandamycins.

Conjugated polyene units also feature in a number of other marine-derived antibacterial natural products. Among the earlier anti-MRSA/anti-VRE compounds to be discovered from marine bacteria were andrimid and moiramides A to C (Figure 18). These metabolites were produced from cultures of a marine isolate of *Pseudomonas fluorescens* [76], although andrimid had previously been isolated from cultures of an *Enterobacter* symbiont taken from a terrestrial plant hopper, *Nilaparvata lugens* [77,78]. Studies with andrimid showed activity against MRSA (MIC: 2 μg/mL) and VRE (32 μg/mL) [76]. Interestingly, the congener with the shorter polyene chain, moiramide B, was apparently more potent against both MRSA (0.5 μg/mL) and VRE (4 μg/mL); moiramides A and C were both inactive [76]. A screen to discover new marine actinomycete-derived natural products with activity against VRE recently identified a *Streptomyces* sp. (strain 307-9) that produced a culture broth with promising activity [79]. The major metabolites responsible for the anti-VRE activity were isolated from fermentation extracts and identified as tirandamycins A and B (Figure 19), compounds that had earlier been isolated from the culture broth of terrestrial *Streptomyces* species, *S. tirandis* and *S. flaveolus* [80,81]. Two new members of this dienoyl tetramic acid family, tirandamycins C and D (respectively Figure 19c and d), were recovered from the cultured marine isolate [79]. Assessment of the anti-VRE activity for the four tirandamycins revealed that the antibacterial action was strongly dependent on the structure of the dioxabicyclo[3.3.1]nonane ring system, with MICs of 2.25, >9, 100 and 110 μM for congeners A, D, B and C respectively [79].

**Figure 20:**
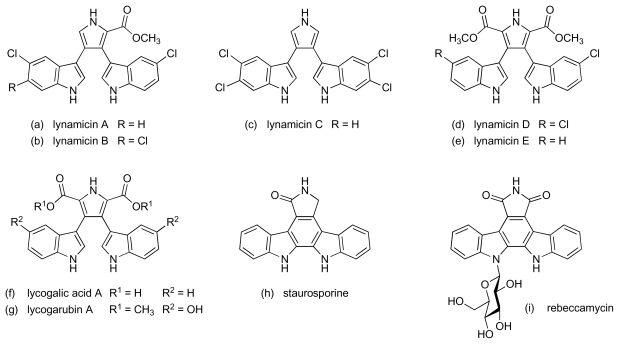
Lynamicins A–E and other bisindole alkaloids.

Bioassay-guided fractionation of the extracts from the culture broth of another *Marinispora* species, designated NPS12745, provided five halogenated bisindole pyrroles, lynamicins A to E (Figure 20a–e), which showed activity against MRSA and VRE [82]. MIC values in the range 1–3 μg/mL and 2–8 μg/mL were recorded for lynamicins A-D against MRSA and VRE, respectively; lynamicin E was somewhat less active (MIC 12 μg/mL and >24 μg/mL against MRSA and VRE) [82]. Bisindole alkaloids, especially those belonging to the bisindolylmaleimide group, such as staurosporine [83] and rebeccamycin [84] (Figure 20h/i), have attracted considerable interest because of their well established antiproliferative activity in cancer cells. In contrast, the bisindole pyrroles are a far less well represented group of compounds, although the core structure is known in metabolites isolated from *Chromobacterium violaceum* and *Lycogala epidendrum* – lycogalic acid A and the lycogarubins (Figure 20f/g) [85–87]. Antibacterial activity for these latter compounds has not previously been reported, however, and it is also thought that the indole ring chlorination status may represent a new chemotype driven by adaptation to the marine environment [82].

**Figure 21:**
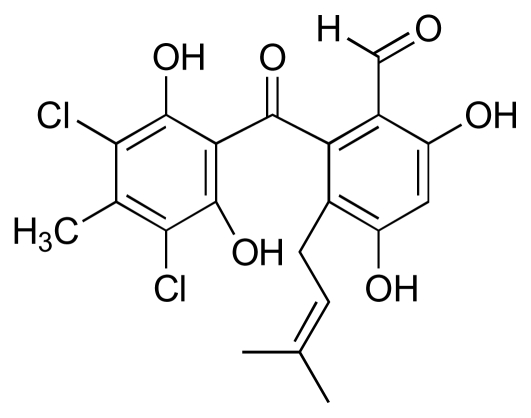
Structure of pestalone.

This review has focused on compounds derived from marine bacteria that exhibit anti-MRSA and anti-VRE activity. Marine fungi are also potentially a rich source of bioactive natural products. Illustrative of this is the benzophenone antibiotic, pestalone (Figure 21), produced in the mixed fermentation of a marine fungus, *Pestalotia* sp. (strain CNL-365) and an unidentified, antibiotic-resistant marine bacterium (CNJ-328) [48]. Pestalone exhibited potent activity against MRSA (MIC: 37 ng/mL) and VRE (MIC: 78 ng/mL). Significantly, pestalone was only produced by the fungus when co-cultured with CNJ-328, highlighting the complex dependence of metabolite biosynthesis on culture conditions and the potential for enhanced antibiotic production through cross-species induction [88–90].

## 3. Conclusions

Although only approximately 3000 compounds have been characterised from marine bacteria, there are already a number of promising compounds demonstrating high levels of activity against MRSA and VRE. Compounds derived from actinomycetes are especially prevalent among those identified in this review. Terrestrial actinomycetes, taken mainly from soil, have provided a rich source of bioactive compounds for more than five decades, yielding in excess of 100,000 bioactive compounds, including 70% of the antibiotics discovered from natural sources [90]. The paucity of new antibiotic classes to emerge since the late 1960s and the challenge now presented by antibiotic-resistant infectious diseases underlines the importance of discovering new chemical diversity from natural sources. Further investigation may prove marine bacteria to be one such important source of compounds with useful clinical applications for recalcitrant and nosocomial infections, such as those caused by MRSA and VRE. Adaptation to the marine environment may provide a driving force for chemical diversity whereas the competitive environment of some marine niches, as with epiphytic bacteria associated with algal surfaces [91], may promote evolutionary development of species capable of producing antibacterial metabolites. Clearly, the recovery of a micro-organism from the ocean does not necessarily imply that it is truly ‘marine’, as some organisms may be wash-in components from the terrestrial environment. Nevertheless, the marine environment with its novel microflora remains a comparatively untapped source of bioactive molecules. Adoption of innovative techniques such as coculturing, cross species induction [92] and biofilm development [89,93], to name three examples, may further facilitate the discovery of new and useful antibiotics.
